# Sickle Cell Disease Subjects Have a Distinct Abnormal Autonomic Phenotype Characterized by Peripheral Vasoconstriction With Blunted Cardiac Response to Head-Up Tilt

**DOI:** 10.3389/fphys.2019.00381

**Published:** 2019-04-11

**Authors:** Patjanaporn Chalacheva, Roberta M. Kato, Payal Shah, Saranya Veluswamy, Christopher C. Denton, John Sunwoo, Wanwara Thuptimdang, John C. Wood, Jon A. Detterich, Thomas D. Coates, Michael C. K. Khoo

**Affiliations:** ^1^Department of Biomedical Engineering, University of Southern California, Los Angeles, CA, United States; ^2^Divisions of Pulmonology, Children’s Hospital Los Angeles, Los Angeles, CA, United States; ^3^Hematology Section, Children’s Center for Cancer, Blood Disease and Bone Marrow Transplantation, Children’s Hospital Los Angeles, Keck School of Medicine of University of Southern California, Los Angeles, CA, United States; ^4^Divisions of Cardiology, Children’s Hospital Los Angeles, Los Angeles, CA, United States

**Keywords:** sickle cell anemia, autonomic dysfunction, orthostatic stress, phenotypic response, peripheral vasoconstriction

## Abstract

In sickle cell disease (SCD), prolonged capillary transit times, resulting from reduced peripheral blood flow, increase the likelihood of rigid red cells entrapment in the microvasculature, predisposing to vaso-occlusive crisis. Since changes in peripheral flow are mediated by the autonomic nervous system (ANS), we tested the hypothesis that the cardiac and peripheral vascular responses to head-up tilt (HUT) are abnormal in SCD. Heart rate, respiration, non-invasive continuous blood pressure and finger photoplethysmogram (PPG) were monitored before, during, and after HUT in SCD, anemic controls and healthy subjects. Percent increase in heart rate from baseline was used to quantify cardiac ANS response, while percent decrease in PPG amplitude represented degree of peripheral vasoconstriction. After employing cluster analysis to determine threshold levels, the HUT responses were classified into four phenotypes: (CP) increased heart rate and peripheral vasoconstriction; (C) increased heart rate only; (P) peripheral vasoconstriction only; and (ST) subthreshold cardiac and peripheral vascular responses. Multinomial logistic regression (MLR) was used to relate these phenotypic responses to various parameters representing blood properties and baseline cardiovascular activity. The most common phenotypic response, CP, was found in 82% of non-SCD subjects, including those with chronic anemia. In contrast, 70% of SCD subjects responded abnormally to HUT: C-phenotype = 22%, P-phenotype = 37%, or ST-phenotype = 11%. MLR revealed that the HUT phenotypes were significantly associated with baseline cardiac parasympathetic activity, baseline peripheral vascular variability, hemoglobin level and SCD diagnosis. Low parasympathetic activity at baseline dramatically increased the probability of belonging to the P-phenotype in SCD subjects, even after adjusting for hemoglobin level, suggesting a characteristic autonomic dysfunction that is independent of anemia. Further analysis using a mathematical model of heart rate variability revealed that the low parasympathetic activity in P-phenotype SCD subjects was due to impaired respiratory-cardiac coupling rather than reduced cardiac baroreflex sensitivity. By having strong peripheral vasoconstriction without compensatory cardiac responses, P-phenotype subjects may be at increased risk for vaso-occlusive crisis. The classification of autonomic phenotypes based on HUT response may have potential use for guiding therapeutic interventions to alleviate the risk of adverse outcomes in SCD.

## Introduction

Sickle cell disease (SCD) is an inherited hemoglobin disorder characterized by transformation of flexible biconcave disk shaped red blood cells into rigid sickle shaped cells caused by polymerization of the abnormal hemoglobin-S once oxygen is released into tissue (Rees et al., 2010). These rigid sickle cells can obstruct microvascular blood flow. Subsequent regional blood flow obstructions can clinically manifest as vaso-occlusive crisis (VOC), resulting in attendant pain, organ damage or death. The mechanism that triggers the transformation from steady-state to VOC remains elusive and the frequency of crises is highly variable among patients. However, onset of VOC events are often associated with emotional stress, cold exposure and pain (Coates et al., 2018), all of which can alter the balance in autonomic nervous system (ANS) activity. In 1976, Eaton et al. (1976) proposed that VOC was triggered by events that prolong red cell transit time in the microvasculature because sickle hemoglobin polymerization would occur in smaller vessels where entrapment was likely. It is known that ANS plays a major role in the regulation of blood flow as blood vessels, particularly arterioles, are innervated with sympathetic neurons (Thomas, 2011). So, abnormal autonomic control of peripheral vascular resistance may predispose SCD patients to prolonged vasoconstriction in response to stressful stimuli. Without compensatory changes in cardiac output, this increases the chance of microvascular blood flow obstruction and VOC.

To date, there is growing evidence of abnormal ANS function in SCD. The interest in autonomic dysfunction in SCD stemmed in part from the increased risk of sudden death in this population (Coates et al., 2018; James et al., 1994; Mestre et al., 1997). Decreased beat-to-beat cardiac variability is common in SCD. Low cardiac variability is a marker of autonomic dysfunction and was found to be a significant predictor of mortality after acute myocardial infarction in non-SCD patients (Kleiger et al., 1987). Our group previously showed that SCD subjects had marked parasympathetic withdrawal in response to transient hypoxia (Sangkatumvong et al., 2011). Other studies found that decreased parasympathetic activity was associated with higher frequency of painful VOC (Nebor et al., 2011) and reported increased sympathetic activity in SCD during VOC compared to their steady state (Charlot et al., 2017). While many studies have employed heart rate variability (HRV) analysis to assess autonomic function, HRV provides information that is directly representative of only cardiac autonomic activity but not peripheral vascular control. In fact, there is a dearth of studies exploring peripheral vascular function in SCD patients. We previously found that SCD subjects had higher frequency of sympathetically-mediated sigh-vasoconstriction (Sangkatumvong et al., 2011) and subsequently, others found that SCD children had stronger vasoconstriction in response to inspiratory breath hold (L’Esperance et al., 2013).

Head-up tilt (HUT) is a potent sympathetic stimulus that triggers both cardiac and peripheral responses and has long been used to assess autonomic function in the context of postural syncope (Zaqqa and Massumi, 2000; Stewart, 2012). During HUT, orthostatic stress causes transient hemodynamic changes which are restored by rapid cardiovascular adjustments through sympathetic activation and parasympathetic withdrawal. As one assumes the upright posture, blood initially shifts toward abdomen and legs, leading to transient drop in stroke volume, cardiac output and subsequently blood pressure. Cardiac and peripheral vascular baroreflexes then act to increase heart rate and peripheral resistance, restoring blood pressure.

In this study, we used HUT as the means to provide an all-encompassing assessment of cardiac and/or peripheral autonomic function in normal controls, SCD subjects and non-SCD subjects with chronic anemia. We hypothesized that by identifying different categories of HUT response among these subjects, we would be able to isolate the autonomic phenotypes that might place SCD subjects at increased risk for microvascular occlusion and VOC. We then employed the causal modeling approach, which utilizes signal analysis and system identification techniques, to probe and disentangle the functional mechanisms involved in the cardiovascular control system (Xiao et al., 2005; Batzel et al., 2009; Khoo, 2018). These advanced analyses provided additional clues into which mechanisms were responsible for predisposing SCD subjects to having different HUT response phenotypes, thereby enabling us to identify the source of the autonomic abnormality in SCD most likely to increase risk of VOC.

## Materials and Methods

### Participants

All experiments were conducted at Children’s Hospital Los Angeles (CHLA). The study protocol was approved by the Committee on Clinical Investigations, the institutional review board (IRB) of CHLA. Participants, who were at least 13 years old, were selected from ethnically matched family members, SCD subjects and other patients followed in the red cell and hemoglobinopathy program at CHLA. Exclusion criteria were any known acute or chronic illnesses including cardiovascular disease that may compromise subject safety or data integrity, significant sickling symptoms and/or vaso-occlusive crisis less than 4 weeks from the scheduled study and known pregnancy. However, non-SCD subjects with chronic anemia were included in order to help disentangle the effect of having SCD from low hemoglobin level. In accordance with CHLA IRB policies, written informed consent or assent (for subjects < 14 years old) was obtained before participation in the study. In addition, parental consent was obtained if the subject was less than 18 years old. Data acquired from total of 66 subjects were studied. Subject characteristics are summarized in Table 1.

**Table 1:** Subject characteristics and baseline physiological measurements.

	Non-SCD (*N* = 39)	SCD (*N* = 27)	*P*-value
Diagnosis	Healthy 11	Homozygous SS 25	–
	Sickle cell trait 8	S-β0 thalassemia 1	
	Hereditary spherocytosis 7	S-β+ thalassemia 1	
	Beta thalassemia major 4		
	Hemoglobin H 5		
	Hemoglobin H constant spring 4		
Age (years)	22.0 (1.2)	23.6 (1.5)	0.40
Sex (M/F)	19/20	13/14	0.96^†^
BMI (kg/m^2^)	24.7 (1.0)	22.6 (0.9)	0.13
Hemoglobin (g/dL)	12.2 (0.4)	9.5 (0.3)	**<0.0001**
Hematocrit (%)	37.2 (0.94)	27.7 (1.03)	**<0.0001**
Reticulocyte^∗^ (%)	1.59 (2.97)	9.02 (9.86)	**<0.0001**
Hemoglobin S^‡^ (%)	–	57 (5.6)	–
Plasma hemoglobin^∗^ (mg/dL)	38.3 (59.8)	71.9 (63.5)	**0.0019**
Free heme^∗^ (μM)	0.18 (1.69)	1.26 (1.23)	**0.0016**
Hemopexin^∗^ (μg/mL)	451 (481)	310 (333)	0.14
SpO_2_^∗^ (%)	98 (0.0)	97 (2)	**<0.0001**


*Normally distributed data are shown as mean (standard error of mean) with p-value from t-test. Non-normally distributed data, indicated by ^∗^, are shown as median (interquartile range) with p-values from Wilcoxon test. ^†^indicates chi-squared test. Bolded p-values indicate statistical significance (p < 0.05). ^‡^Hemoglobin S that can polymerize under normal conditions such as in SCD patients, unlike in sickle cell trait patients.*

### Protocols and Data Preprocessing

Subjects were asked to stay hydrated and get adequate sleep the day before the study and avoid caffeinated beverages on the day of the study. The study was always carried out in the morning, starting between 9 and 11 am. The study took place in the autonomic lab, a quiet, dimmed light and temperature-controlled room. After the subject had rested quietly for at least 5 min, we recorded heart rate, blood pressure and oxygen saturation (SpO_2_). Then up to 30 ml of blood was drawn for complete blood count, reticulocyte count, hemoglobin electrophoresis, plasma hemoglobin, plasma free heme and hemopexin.

The subject was positioned on the tilt table. Before the actual study protocol started, the subjects underwent a short HUT (<1 min) to familiarize them with the protocol as well as to let them position themselves properly as they transitioned from supine to 70° upright position. The subjects were then returned to supine position. After all vital signals stabilized, we began collecting baseline (no intervention) data for 7 min. The subjects underwent a battery of autonomic challenges, such as induced hypoxia, handgrip and controlled breathing, which were not related to the HUT protocol presented in this paper. Each of these challenges, including HUT, was separated by a washout period. The HUT protocol started with 5-min pre-HUT recording in supine position. The subject was then tilted up to 70° upright position at a rate of approximately 5°/s and remained in that position for 7 min before being returned to supine. Following stabilization of all signals, recording continued post-HUT for at least 5 min.

The electrocardiogram (ECG), continuous blood pressure (Nexfin; BMEYE, Amsterdam, Netherlands), photoplethysmogram (Nonin Medical Inc., United States), and respiratory airflow using a pneumotachometer (Hans Rudolph, Inc., Kansas City, MO, United States) were monitored. Blood pressure and photoplethysmogram (PPG) were measured on the index finger and the thumb on the right hand, respectively. Cutaneous blood flow using laser Doppler flowmetry (Perimed, Jarfalla, Sweden) was also measured on the right ring finger for consistency checks with the PPG measurements but was not used in the analyses (see section “Discussion”). In addition, an accelerometer was attached to the tilt table to capture the exact moment when HUT occurred. All measurements were digitized synchronously and continuously through Biopac MP150 data acquisition system (Biopac, United States) at 250 Hz. The beat-to-beat variables were extracted in relation to the R-waves on the ECG. R–R interval (RRI) was defined as the time between two consecutive R-waves. Diastolic and systolic blood pressure (DBP and SBP) were the nadir and the peak of the blood pressure pulse within each RRI. The mean arterial pressure (MAP) for each beat was calculated from the average of the continuous blood pressure values over the cardiac cycle. The pulse amplitude of the PPG (PPGa) was derived as the difference between the peak and nadir of PPG signal within each beat. PPGa reflects pulsatile change in finger blood volume caused by arterial blood flow in the fingertip (Allen, 2007; Elgendi, 2012) and is related to blood flow and arterial compliance (Beene and Eggers, 1974; Babchenko et al., 2001). The modulation of arterial compliance is primarily governed by the sympathetic nervous system as previous studies have demonstrated that PPGa increases significantly during sympathetic blockade (Beene and Eggers, 1974; Kim et al., 1975; Babchenko et al., 2001). As such, we took the decreases/increases in PPGa to represent vasoconstriction/dilation in response to neural inputs. Since PPGa is a relative measurement, it was normalized to its own 95th percentile value of its full study recording and expressed in normalized unit (nu). Lastly, the respiratory airflow was integrated to produce instantaneous lung volume (ILV) change.

For subsequent spectral and modeling analyses, all beat-to-beat variables, namely RRI, SBP, DBP, MAP, and PPGa, and the corresponding respiration signal (ILV), were converted into uniformly sampled time series, with 0.5 s as the interval between samples, using interpolation and resampling algorithm of Berger et al. (1986).

### HUT Response Quantification

Tachycardia and peripheral vasoconstriction are the compensatory responses to transient drop in blood pressure during HUT. We defined the cardiac response (decrease in mean RRI) and peripheral response (decrease in mean PPGa) as percent change relative to their own supine values:

(1) $$\Delta \text{ RRI or }\Delta \text{ PPGa=}\left(\frac{\text{mean supine-mean HUT}}{\text{mean supine}}\times 100\right)$$

**FIGURE 1:**
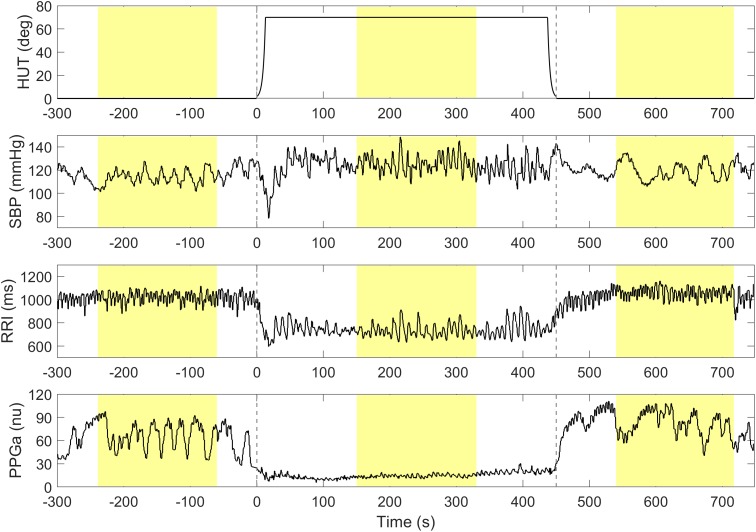
A representative data segment pre-, during and post-HUT. Vertical dashed lines indicate the onset and the end of HUT. The mean values of SBP, RRI and PPGa during pre-, during and post-HUT are calculated from the shaded areas.

Higher ΔRRI and ΔPPGa signified stronger tachycardic and vasoconstrictive responses, respectively. To avoid including transient HUT-related responses, the mean values of RRI, PPGa, and SBP were derived from 3-min during the pre-HUT period at least 60 s before HUT onset, during HUT 150 s after HUT onset, and during post-HUT 90 s after returning to supine (Figure 1). We chose the post-HUT means rather than pre-HUT values to be the reference supine values for RRI and PPGa as they were more consistent with the baseline recorded at the very beginning of the entire study session (see Supplementary Materials). Mean SBP was carefully monitored to determine whether the subjects could restore their blood pressure level during HUT.

### HUT Response Classification

**FIGURE 2:**
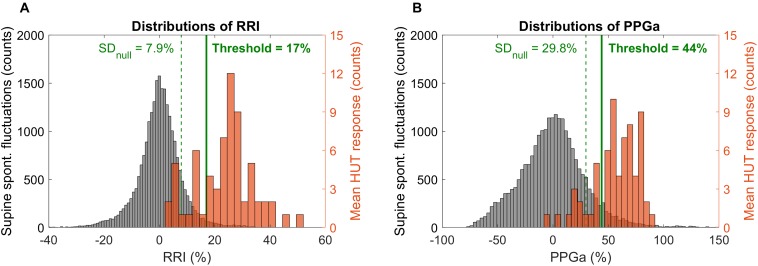
Distributions of spontaneous fluctuations during supine (null distributions, gray) and distributions of the mean change during HUT (orange) in RRI **(A)** and PPGa **(B)**. Note that the distributions of spontaneous fluctuations consider all sample points during supine while the mean HUT response distributions constitute of one value of ΔRRI or ΔPPGa per subject. Dashed vertical lines indicate 1 standard deviation of the null distributions (level of spontaneous fluctuations during supine). Solid lines indicate the optimal thresholds in RRI and PPGa for HUT response classification.

Determination of thresholds for response to HUT was first done by examination of the frequency distributions of percent change in RRI and PPGa in response to HUT. The RRI and PPGa data during both supine and HUT were expressed as percent change relative to the subject’s own supine value. The null distributions for fluctuations in RRI and PPGa while supine (expressed in %) were constructed for all subjects, reflecting the “population” spontaneous fluctuations in RRI and PPGa. The subject was considered as having a substantial HUT response (i.e., the responses exceeded spontaneous fluctuations) if the ΔRRI or ΔPPGa during HUT (expressed in %) was greater than 1 standard deviation (SD) of the supine null distribution (Figure 2, dashed lines). Based on a cutoff at 1 SD, the subjects were initially classified into four groups (combinations of having ΔRRI and ΔPPGa during HUT above or below the thresholds).

**FIGURE 3:**
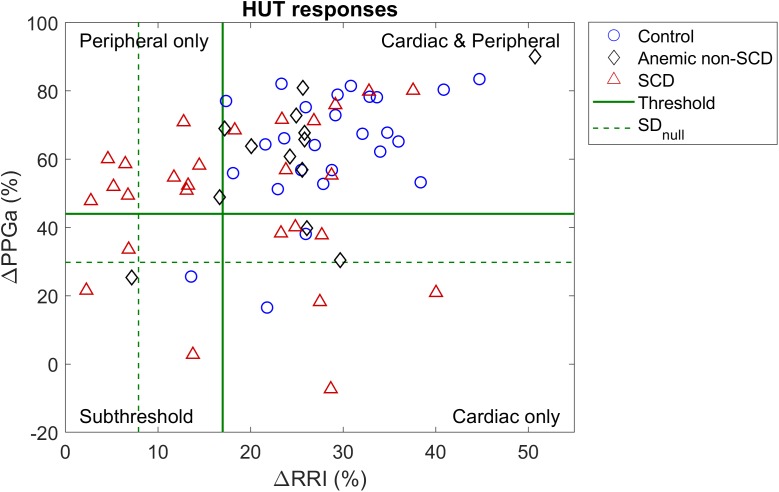
Scatter plot of percent mean change in RRI and PPGa during HUT relative to supine value. Dash lines (SD_null_) represent the level of spontaneous fluctuations during supine in RRI and PPGa. Solid lines are the optimal thresholds that divide subjects into clusters of different HUT response phenotypes. Most subjects were classified into the dual cardiac and peripheral response phenotype (CP, upper right quadrant). However, the peripheral vasoconstriction only phenotype (P, upper left quadrant) consisted of mainly SCD subjects.

**FIGURE 4:**
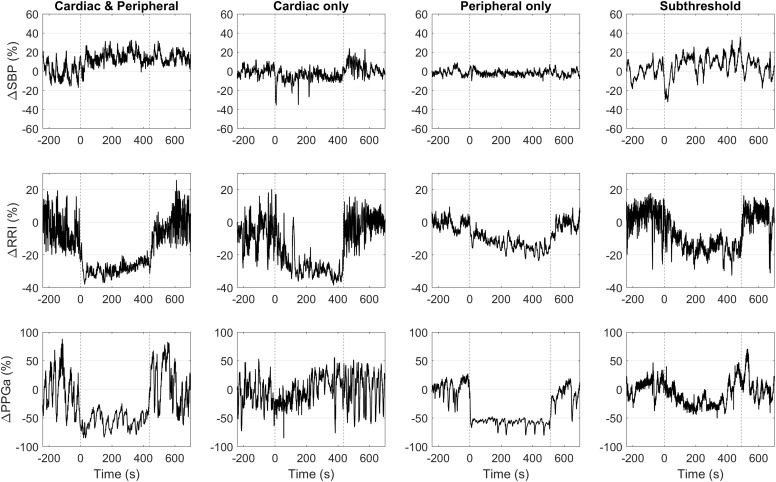
Representative data of four subjects with different HUT response phenotypes. Vertical dashed lines mark the onset and the end of HUT.

These thresholds were further refined by first visualizing ΔRRI and ΔPPGa of each subject as a data pair. The scatter plot of ΔRRI vs. ΔPPGa is shown in Figure 3. Next, we searched for the RRI and PPGa thresholds that best separated the data pairs into four quadrants (i.e., four groups) by minimizing the dispersion of data pairs from the center of each group (Everitt et al., 2011). We allowed the RRI and PPGa thresholds to vary from 1 to 3 SDs. One SDs of RRI and PPGa were selected as the minimal thresholds to ensure that the responses at least exceeded spontaneous fluctuations. The combination of these thresholds that yielded the minimal total dispersion of data pairs in all groups was selected as the optimal cutoff RRI and PPGa thresholds. We used these final thresholds to classify subjects into 4 phenotypic responses to SBP recovery during HUT: (1) dual cardiac rate and peripheral vasoconstriction responses (CP), (2) only cardiac rate response (C), (3) only peripheral vasoconstriction response (P) and (4) subthreshold cardiac rate and peripheral vasoconstriction responses (ST). SBP, RRI, and PPGa of representative subjects with different HUT responses are shown in Figure 4.

### Baseline Physiological and Autonomic Parameters

To assess how the individuals’ baseline physiological characteristics affected their HUT responses, we calculated mean RRI, SBP, DBP, and PPGa and autonomic indices from their corresponding study baseline. The autonomic indices were derived from heart rate variability, blood pressure variability and peripheral vascular variability as follows:

(1) Normalized high-frequency power of RRI (HFP_RRI,n_): RRI spectral power in 0.15–0.4 Hz region divided by the square of mean RRI, representing parasympathetic modulation of RRI (ESC/NASPE Task Force, 1996; Sacha, 2013).
(2) Low-to-high ratio (LHR_RRI_): ratio of low-frequency RRI spectral power (0.04–0.15 Hz) to high-frequency RRI spectral power, representing sympatho-vagal balance (ESC/NASPE Task Force, 1996).
(3) Low-frequency power of SBP (LFP_SBP_): SBP spectral power in 0.04–0.15 Hz region, representing sympathetic modulation on blood pressure (Parati et al., 1995; Pagani et al., 1996; Malpas, 2002).
(4) Baroreflex sensitivity (BRS): determined using the sequence method (Parati et al., 2000), reflecting how much RRI changes in response to spontaneous changes in SBP.
(5) Low frequency power of PPGa (LFP_PPGa_): PPGa spectral power in 0.04–0.15 Hz region, reflecting vascular variability in response to stimuli such as sympathetic nerve inputs.

The spectral powers were computed using the Welch method (Mitra, 2006). These physiological and autonomic parameters, along with subject characteristics, were then used to predict the HUT response phenotypes in SCD and non-SCD subjects using multinomial logistic regression (MLR) analysis. Details of the MLR analysis are described in the Statistical tests section.

### Model-Derived Autonomic Indices

To further explore what functional mechanisms might account for the differences in baseline autonomic characteristics among HUT response phenotypes, we employed an input-output modeling approach to capture the functional dependencies among the various key physiological variables (Khoo, 2018). The mathematical model allows us to disentangle the effect of each model input, e.g., blood pressure and respiration, on the model output, e.g., heart rate and vascular responses. An important feature of this approach is that the model can have multiple inputs but the dynamics between each input and the output have to be “causal” – i.e., changes in the output at the present time can only be ascribed to changes in one or more of the inputs that occurred in the past. Based on our understanding of the underlying physiology, we assumed that fluctuations in RRI (δ*RRI*) are derived from two main mechanisms: the arterial baroreflex (ABR), relating changes in SBP (δ*SBP*) to δRRI, and respiratory-cardiac coupling (RCC). RCC could represent a broad variety of physiological mechanisms through which respiration can affect heart rate. These include central respiratory entrainment of cardiovagal traffic from the medulla, vagal feedback from the pulmonary stretch receptors, mechanical stretching of the sinoatrial node, and contributions from the cardiopulmonary reflexes due to respiratory changes in venous return (Lucini et al., 2000; Belozeroff et al., 2003). The mathematical representation of the model of heart rate variability is

(2) $$\delta RRI(t)=\overset{M-1}{\underset{i=0}{\Sigma }}h_{ABR}(i)\cdot \delta SBP(t-i-T_{ABR})+\overset{M-1}{\underset{i=0}{\Sigma }}h_{RCC}(i)\cdot \delta ILV(t-i-T_{RCC})+\varepsilon_{RRI}\left(t\right)$$

In similar fashion, peripheral vascular variability was assumed to be derived from two main mechanisms: blood pressure-peripheral vascular coupling (BPC) and the respiratory-peripheral vascular coupling (RPC) (Chalacheva and Khoo, 2014; Khoo and Chalacheva, 2016). BPC relates the fluctuations in mean arterial blood pressure (δ*MAP*) to fluctuations in PPGa (δ*PPG*_a_) through sympathetically mediated baroreflex control of peripheral resistance (Guyenet, 2006) and through local regulation of blood flow (Davis and Hill, 1999; Secomb, 2008). RPC relates changes in respiration (δ*ILV*) to PPGa fluctuations through respiratory modulation of sympathetic neural activity, which in turn affects peripheral vascular resistance (Seals et al., 1993; Malpas et al., 2001). The mathematical representation for peripheral vascular variability is:

(3) $$\delta PPGa(t)=\overset{M-1}{\underset{i=0}{\Sigma }}h_{BPC}(i)\cdot \delta MAP(t-i-T_{BPC})+\overset{M-1}{\underset{i=0}{\Sigma }}h_{RPC}(i)\cdot \delta ILV(t-i-T_{RPC})+\varepsilon_{PPGa}\left(t\right)$$

Equations (2) and (3) describe how δ*RRI* and δ*PPGa* at the current time, represented by sample index *t*, are influenced by the cumulative effects of past (up to *M* + latency *T*) values of δ*SBP*, δ*MAP*, and δ*ILV*. *h*_ABR_, *h*_RCC_, *h*_BPC_, and *h*_RPC_ represent the “standardized” RRI or PPGa responses to unit pulse increases in the corresponding inputs. These are also known as the “impulse responses” (Khoo, 2018). *M* denotes the system memory or the time (number of samples) it takes for an impulse response to decay to zero. ε_RRI_ and ε_PPGa_ represent the model residuals, the extraneous influences on RRI or PPGa fluctuations that are not accounted for by the models. *T*_ABR_, *T*_RCC_, *T*_BPC_, *T*_RPC_ denote the latencies (in number of samples) associated with the corresponding functional mechanisms.

The above mathematical models were applied to data collected from all SCD subjects during the baseline period to quantify the underlying autonomic characteristics for each of the subjects. The impulse responses were estimated using the basis function expansion technique (Khoo and Chalacheva, 2016), where the impulse response was represented as a weighted sum of a set of basis functions as follows

(4) $$h_{x}(t)=\overset{q_{x}}{\underset{i=1}{\Sigma }}c_{x}(i)B_{i}^{\left(n_{x}\right)}\left(t\right)$$

*h*_x_ represents the impulse response relating input *x* to the output. In this case, *x* represents δ*SBP*, δ*MAP*, or δ*ILV*. *q*_x_ is the number of basis functions used in the expansion of an impulse response. *B_i_*^(*n*)^ (*t*) represents the orthonormal sets of Meixner functions with *n*th order of (Asyali and Juusola, 2005). *c*_x_ represents each of the expansion coefficients of the basis functions for the impulse response. Further details of the procedures for solving Equations (2), (3), and (4) to estimate the impulse responses may be found in Belozeroff et al. (2003) and Khoo (2018).

Once the impulse responses of these mechanisms were obtained, the transfer functions were determined by taking the Fourier transforms of the estimated impulse responses. The average transfer function magnitudes in the high-frequency (0.15–0.4 Hz) region, ABR_HF_ and RCC_HF_, were calculated and taken to represent the gains through which SBP fluctuations and respiration, respectively, contribute to parasympathetic modulation of RRI (i.e., HFP_RRI_). Similarly, the average transfer function magnitudes in the low-frequency (0.04–0.15 Hz) region, BPC_LF_ and RPC_LF_, were computed and taken to represent the gains with which SBP fluctuations and respiration, respectively, contribute to sympathetic modulation of fluctuations in PPGa. Previous studies by us (Belozeroff et al., 2003) and others (Lucini et al., 2000) have shown that ABR_HF_ correlates well with the more broadly used measure of BRS derived from the sequence technique. However, an important difference between the model-based method and sequence technique for assessing baroreflex gain is that estimates of ABR_HF_ are obtained after computationally adjusting for the direct influence of respiration [see Equation (2) above].

### Statistical Tests

Student’s *t*-test (or Wilcoxon test if data were not normally distributed) was used to test for differences in subject characteristics between SCD and non-SCD subjects. Paired *t*-test was used to compare the SBP values to determine if these subjects could restore their blood pressure level during HUT.

The relation among subject characteristics, baseline autonomic indices and the HUT response phenotypes were examined in two stages by multinomial logistic regression (MLR) analysis (Domínguez-Almendros et al., 2011). Candidate variables for the MLR were included if *p* < 0.20 in the univariate analysis. Log-transformation was applied to highly skewed variables to satisfy assumptions of logistic regression. The selected candidate variables were entered in a stepwise regression. If the candidate variables were correlated with each other (e.g., hemoglobin, plasma free hemoglobin and free heme), the variable were added to the model one at a time to avoid multicollinearity. Finally, a set of significant predictors constituted the final MLR model. For all MLR analyses, we let the CP phenotype be the reference category for the HUT response phenotypes. Using the final MLR model, we also calculated the predicted probability of falling into different HUT response phenotypes as each covariate varied at different levels: low (10th percentile), moderate (median) and high (90th percentile), to investigate the effect of each covariate while controlling for other factors, i.e., holding other parameters at their median values.

Analysis of variance (ANOVA) was used to test the model-derived autonomic indices of SCD subjects for differences among HUT response phenotypes. If the variables were not normally distributed, the variables were log-transformed to satisfy the ANOVA assumption (Shapiro–Wilk normality test, assuming normality if *p* > 0.05). Dunnett’s test was applied *post hoc* if ANOVA detects significant difference among phenotypes with the CP-phenotype being the control group.

For all statistical tests, the statistical significance was defined as *p* < 0.05. All statistical analyses were performed using JMP statistical software, version 13.0 (SAS Institute Inc., Cary, NC, United States).

## Results

### Subject Characteristics

Tables 1, 2 summarize subject characteristics and baseline physiological and autonomic indices. There was no difference in age, sex, body mass index (BMI), mean baseline RRI, SBP, DBP, PPGa or any autonomic indices between SCD and non-SCD subjects. However, SCD subjects had lower hemoglobin, hematocrit and SpO_2_, and higher reticulocyte count, plasma free hemoglobin and free heme than non-SCD subjects.

**Table 2:** Baseline physiological and autonomic indices.

	Non-SCD	SCD	*P*-value
RRI (ms)	906 (22)	875 (28)	0.37
SBP (mmHg)	117 (1.8)	113 (2.2)	0.20
DBP (mmHg)	68.5 (1.5)	66.0 (1.3)	0.23
PPGa (nu)	66.4 (3.0)	66.0 (3.7)	0.93
HFP_RRI,n_^∗^ (nu/Hz)	0.00187 (0.0020)	0.00178 (0.0028)	0.81
LHR_RRI_^∗^ (unitless)	0.40 (0.34)	0.48 (0.80)	0.12
LFP_SBP_^∗^ (mmHg^2^/Hz)	6.83 (7.48)	4.89 (6.23)	0.22
BRS^∗^ (ms/mmHg)	17.2 (11.4)	15.0 (12.9)	0.32
LFP_PPGa_^∗^ (nu)	35.7 (47.9)	54.1 (47.8)	0.27


*Normally distributed data are shown as mean (standard error of mean) with p-value from t-test. Non-normally distributed data, indicated by ^∗^, are shown as median (interquartile range) with p-values from Wilcoxon test. RRI, R-R interval; SBP, systolic blood pressure; DBP, diastolic blood pressure; PPGa, photoplethysmogram amplitude; HFP_*RRI,n*_, normalized high-frequency power of RRI; LHR_*RRI*_, low-to-high ratio of RRI; LFP_*SBP*_, low-frequency power of SBP; BRS, baroreflex sensitivity; LFP_*PPGa*_, low-frequency power of PPGa.*

### HUT Responses

In most patients there was an initial drop in SBP at the onset of tilt; there was no significant difference (*p* = 0.1961) between pre-HUT SBP (119 mmHg) and during HUT SBP (117 mmHg), suggesting that most subjects were able to restore their blood pressure during HUT. However, there were 5 subjects with >20% drop in SBP during HUT from their pre-HUT SBP: 3 from the CP group, 1 from the P group and 1 from the ST group. None of the subjects showed or reported any signs of syncope.

The scatter plot of ΔRRI (cardiac response to HUT) vs. ΔPPGa (peripheral vascular response to HUT) is shown in Figure 3. The spontaneous fluctuations in RRI and PPGa during supine were 7.9 and 29.8%, respectively (dashed lines, denoted as SD_null_). The optimal thresholds that best separate subjects into four HUT phenotypes were 17 and 44% for RRI and PPGa, respectively (solid lines). Table 3 tabulates the number of SCD and non-SCD subjects in different HUT groups. Most subjects were classified as having dual cardiac and peripheral vascular responses to HUT; however, subjects with only peripheral vasoconstriction response were primarily patients with SCD (likelihood ratio χ^2^ = 22.4, *p* < 0.0001).

**Table 3:** SCD and non-SCD subjects by HUT response classification: count (row %).

		HUT phenotype				
	CP	C	P	ST	Total
SCD	8 (29.6)	6 (22.2)	10 (37.0)	3 (11.1)	27
Non-SCD	32 (82.1)	4 (10.3)	1 (2.6)	2 (5.1)	39
Total	40	10	11	5	66


*The contingency table shows the number of SCD and non-SCD subjects in different HUT phenotypes (likelihood ratio χ^*2*^ = 22.4, p < 0.0001). CP, having both cardiac and peripheral responses; C, having only cardiac response; P, having only peripheral response; ST, having subthreshold cardiac and peripheral response.*

### Effects of Baseline Characteristics on HUT Responses

Univariate associations predicting the response to HUT were examined and the potential candidates for stepwise regression analysis were: diagnosis, age, sex, SpO_2_, hemoglobin, hematocrit, reticulocyte, free heme, baseline DBP, PPGa, HFP_RRI,n_, LFP_SBP_, BRS and LFP_PPGa_ (Table 4). Stepwise regression analysis selected the following as the covariates in the final MLR model of HUT response phenotypes: diagnosis, hemoglobin, baseline HFP_RRI,n_ and baseline LFP_PPGa_ (*p* < 0.0001, χ^2^ = 61.96, DF = 12, *R*^2^ = 0.43). These variables predict 43% of the variance with a misclassification rate of 22% suggesting that this model predicts the HUT response phenotypes with 78% accuracy.

**Table 4:** Univariate analysis for HUT response groups.

Parameters	*P*-value
**Diagnosis**	**<0.0001**
**Age**	**0.1674**
**Sex**	**0.1725**
Height	0.26
Weight	0.37
BMI	0.21
**SpO_2_**	**0.0034**
**Hemoglobin**	**0.0004**
**Hematocrit**	**0.0002**
**Reticulocyte^∗^**	**0.0328**
Plasma hemoglobin^∗^	0.21
**Free heme^∗^**	**0.0569**
Hemopexin^∗^	0.93
Baseline RRI	0.23
Baseline SBP	0.24
**Baseline DBP**	**0.0416**
**Baseline PPGa**	**0.0077**
**Baseline HFP_RRI,n_^∗^**	**0.0002**
Baseline LHR_RRI_^∗^	0.35
**Baseline LFP_SBP_^∗^**	**0.1669**
**Baseline BRS**	**0.0226**
**Baseline LFP_PPGa_^∗^**	**0.1883**


*P-value column shows the significance of each parameter in estimating HUT phenotypes. Bolded parameters are parameters that will enter the stepwise regression analysis (p < 0.20) for selection of the covariate set of the final multinomial regression model. ^∗^indicates log-transformed data. SpO_*2*_, oxygen saturation; RRI, R–R interval; SBP, systolic blood pressure; DBP, diastolic blood pressure; PPGa, photoplethysmogram amplitude; HFP_*RRI,n*_, normalized high-frequency power of RRI; LHR_*RRI*_, low-to-high ratio of RRI; LFP_*SBP*_, low-frequency power of SBP; BRS, baroreflex sensitivity; LFP_*PPGa*_, low-frequency power of PPGa.*

**FIGURE 5:**
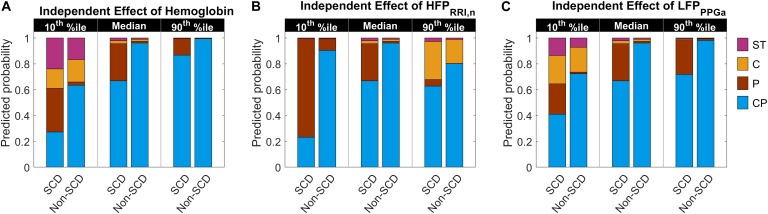
Predicted probability of having certain HUT response phenotype as hemoglobin level, HFP_RRI,n_ and LFP_PPGa_ independently vary from low (10th percentile) to high (90th percentile) level. The middle pair of bars in each subfigure are the same and show the probability when hemoglobin **(A)**, HFP_RRI,n_ **(B)**, and LFP_PPGa_ **(C)** are all at their respective median levels. CP, having dual cardiac and peripheral response; P, having only peripheral response; C, having only cardiac response; and ST, having subthreshold cardiac and peripheral responses to HUT. The height of each colored column in each stacked bar represents the probability of having the corresponding phenotypic response to HUT.

The parameter estimates of the MLR analysis of HUT responses and their corresponding *p*-values are listed in Table 5. We found that SCD subjects were 33 times more likely to have only peripheral vasoconstriction in response to HUT than non-SCD subjects, after controlling for hemoglobin, baseline HFP_RRI,n_ and LFP_PPGa_. Figure 5 summarizes the effect of the independent predictors of HUT response phenotype. The middle pair of bars in Figure 5A–C are the same, and show that the probability of SCD subjects having only peripheral vasoconstriction as a primary mechanism for SBP recovery during HUT (P category) is 29% when hemoglobin, HFP_RRI,n_ and LFP_PPGa_ are all at their respective median levels. When hemoglobin alone is decreased to the 10th percentile (Figure 5A), the probability of having dual response to HUT (CP) is reduced to 27% in SCD and to 64% in non-SCD. In contrast, when the hemoglobin level is at the 90th percentile, almost all SCD and non-SCD subjects have dual response. When HFP_RRI,n_ by itself is lowered to the 10th percentile (Figure 5B), indicating decreased baseline parasympathetic activity, the probability of having dual response is reduced to 23% in SCD while the probability of having only peripheral vasoconstriction response (P) increases to 76%. In contrast, when HFP_RRI,n_ is at the 90th percentile, the probability of having only tachycardic response to HUT (C) increases to 29% in SCD and the probability of having only peripheral vasoconstriction (P) is reduced to 5%. When LFP_PPGa_, reflecting vascular variability at baseline, is at the 10th percentile (Figure 5C), the probability of having subthreshold response (ST) is significantly increased for both SCD and non-SCD with little effect on the probability of having peripheral vasoconstriction only (P). However, when the vascular variability is at the 90th percentile, most subjects have normal dual response to HUT regardless of diagnosis.

**Table 5:** Parameter estimates of the final multinomial logistic regression of HUT phenotypes.

Parameter	HUT phenotypes	Estimate	*SE*	*P*-value
Intercept	C	23.18	7.99	0.0037
Intercept	P	-7.56	6.30	0.23
Intercept	ST	13.03	7.92	0.10
Diagnosis				**0.0082^†^**
Diagnosis [SCD]	C	0.70	1.14	0.54
Diagnosis [SCD]	P	3.50	1.28	**0.0065**
Diagnosis [SCD]	ST	1.21	1.22	0.32
Hemoglobin				**0.0096^†^**
Hemoglobin	C	-0.69	0.32	**0.0322**
Hemoglobin	P	-0.27	0.26	0.31
Hemoglobin	ST	-0.83	0.37	**0.0242**
Baseline HFP_RRI,n_^∗^				**<0.0001^†^**
Baseline HFP_RRI,n_^∗^	C	4.17	1.57	**0.0080**
Baseline HFP_RRI,n_^∗^	P	-2.64	1.28	**0.0394**
Baseline HFP_RRI,n_^∗^	ST	0.49	1.61	0.76
Baseline LFP_PPGa_^∗^				**0.0032^†^**
Baseline LFP_PPGa_^∗^	C	-4.59	1.61	**0.0044**
Baseline LFP_PPGa_^∗^	P	-0.49	1.75	0.78
Baseline LFP_PPGa_^∗^	ST	-3.89	1.72	**0.0243**


*The global multinomial logistic regression model test was significant (χ^*2*^ = 61.96, DF = 12, p < 0.0001). ^∗^indicates log-transformed data. ^†^indicates p-value of the overall effect test (the change in fit resulting from discarding one of the covariates). P-value column shows the significance of each parameter estimate, with CP group being the reference HUT phenotype. Bolded p-values indicate statistical significance (p < 0.05). CP, having both cardiac and peripheral responses; C, having only cardiac response; P, having only peripheral response; ST, having subthreshold cardiac and peripheral response; HFP_*RRI,n*_, normalized high-frequency power of RRI; LFP_*PPGa*_, low-frequency power of photoplethysmogram amplitude.*

### Baseline Characteristics of SCD Subjects With P Phenotype

**FIGURE 6:**
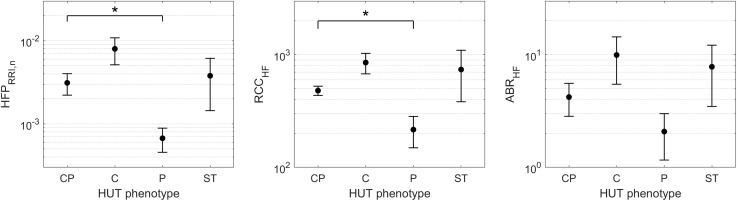
HFP_RRI,n_ and model-derived cardiac autonomic indices of SCD subjects across HUT phenotypes (mean ± SEM). ^∗^indicates significant pairwise difference from the CP phenotype. SCD subjects with the P phenotype had significantly lower baseline parasympathetic activity (HFP_RRI,n_, **left**) than those with typical HUT response (CP phenotype). The mathematical modeling analysis revealed that the low parasympathetic activity in SCD subjects with the P phenotype was due to impaired respiratory-cardiac coupling (RCC_HF_, **middle**). The arterial baroreflex gain (ABR_HF_, **right**) followed similar trend to HFP_RRI,n_ across phenotypes but the difference did not reach statistical significance.

Although the SCD subjects we studied displayed all 4 HUT phenotypes, the P phenotype consisted overwhelmingly of SCD subjects (Figure 3). So what baseline characteristics distinguished the P-phenotype SCD subjects from the other SCD subjects? Based on the MLR analysis, the probability of having the P phenotype in SCD subjects substantially increased if those subjects were to have low baseline parasympathetic activity (Figure 5B). HFP_RRI,n_ was indeed lower in SCD subjects with the P phenotype compared SCD subjects with normal (CP) response (*p* = 0.0144). We used mathematical modeling to delineate which functional mechanisms might account for the low HFP_RRI,n_ in P-phenotype subjects. We found that the P-phenotype SCD subjects had significantly lower high-frequency RCC gain (RCC_HF_) than those with CP phenotype (*p* = 0.010, Figure 6). The high-frequency ABR gain (ABR_HF_) also followed similar trends to those of HFP_RRI,n_ but this parameter was not significantly different from CP phenotype (Table 6). As a consistency check, we found that the BRS across SCD phenotypes agreed with ABR_HF_ and did not show any significant difference from the CP phenotype.

**Table 6:** Model-derived autonomic indices in SCD subjects by HUT phenotype.

	CP	C	P	ST	*P*-value
RCC_HF_ (ms/L)	487 (224)	698 (744)	148 (156)*	451 (1132)	**0.0010**
ABR_HF_ (ms/mmHg)	3.9 (5.7)	6.2 (17.3)	1.7 (3.6)	3.9 (13.4)	0.14
RPC_LF_ (nu/L)	136 (295)	70 (95)	114 (178)	274 (162)	0.13
BPC_LF_ (nu/mmHg)	4.8 (5.2)	2.9 (0.6)	5.4 (2.2)	2.5 (2.3)	0.08


*Data are shown as median (interquartile range) with p-value from ANOVA. Bolded p-values indicate statistical significance (p < 0.05). ^∗^ indicates significant pairwise difference from the CP phenotype (p = 0.010, Dunnett’s test). RCC_*HF*_, high-frequency respiratory-cardiac coupling gain; ABR_*HF*_, high-frequency arterial baroreflex gain; RPC_*LF*_, low-frequency respiratory-peripheral vascular coupling gain; BPC_*LF*_, low-frequency blood pressure-peripheral vascular coupling gain.*

Another significant autonomic predictor of HUT phenotypes was baseline LFP_PPGa_, representing vascular variability. The probability of having the C phenotype increased as LFP_PPGa_ became lower (Figure 5C). This was, however, the case for both SCD and non-SCD subjects. The SCD subgroup analysis showed that there was no difference in LFP_PPGa_ across HUT phenotypes. Consistent with this finding, model-based analysis of the functional mechanisms governing vascular variability confirmed that low-frequency BPC and RPC gains (BPC_LF_ and RPC_LF_), were not different across phenotypes (Table 6).

## Discussion

### Autonomic Responses to Head Up Tilt

The HUT test is commonly used to evaluate orthostatic intolerance (Zaqqa and Massumi, 2000; Stewart, 2012) but it has also been employed to assess autonomic function in various disease states (Chandler and Mathias, 2002; Wang et al., 2012). During HUT, the shift in blood volume from the upper to lower parts of the body leads to a transient drop in blood pressure, triggering the arterial and cardiopulmonary reflexes. These reflexes result in parasympathetic withdrawal, thereby increasing heart rate, and increased sympathetic drive, which increases vascular tone and peripheral resistance, along with heart rate and cardiac contractility. In subjects without orthostatic intolerance, the net effect of all these reflex actions is the maintenance of blood pressure at or close to pre-tilt levels. However, the relative changes in vagal control of heart rate and sympathetic control of vascular resistance and tone within and across individuals have not been systematically studied, and particularly so on a quantitative basis.

In this study, we used PPGa as a surrogate measure of peripheral vascular conductance. Implicit in this is the assumption that the changes in PPGa we measured represent primarily changes resulting from peripheral vasoconstriction/dilation. However, a fraction of the changes in PPGa could also have been due to changes in pulse pressure, secondary to changes in stroke volume during HUT. Pulse pressure was indeed reduced during HUT (∼40.7 mmHg) compared to supine (∼47.5 mmHg). However, in terms of percent change from supine levels, the mean decrease in pulse pressure was 13.1%, substantially smaller than the corresponding 56.7% reduction in PPGa, suggesting that stroke volume played a secondary role in contributing to the decrease in PPGa during HUT. Additional analysis of cutaneous blood flow (based on laser Doppler flowmetry) in the same hand showed that the mean percent decrease in microvascular flow from supine to HUT was 30.5%. Since this estimate was based on beat-averaged values of the flow signal and did not take into account the amplitude of the pulsatile component, it represents changes in mean microvascular flow with minimal direct influence from pulse pressure. This decrease in mean cutaneous blood flow provides further support to the notion that the reduction in PPGa during HUT was mainly due to peripheral vasoconstriction rather than changes in stroke volume.

### Phenotyping the Responses to HUT

In the current study, we measured the responses of SCD, anemic non-SCD and healthy subjects to HUT, and classified them on the basis of the corresponding relative changes in heart rate and peripheral vascular responses. As mentioned in the Section “Materials and Methods,” the responses were divided into four different phenotypes: (1) dual cardiac and peripheral vascular responses (CP), (2) primarily cardiac rate response (C), (3) primarily peripheral vascular response (P) and (4) subthreshold cardiac and peripheral vascular responses (ST). We found that the majority of subjects (∼60%) regardless of diagnosis belonged to the CP phenotype. However, the group with P phenotype consisted predominantly of SCD subjects. This remained true even after adjusting for the effects of anemia. In SCD subjects, low cardiac parasympathetic activity at baseline dramatically increased the probability of having P-phenotype response (Figure 5B). This suggests there is a characteristic autonomic dysfunction that is unique to subjects with SCD compared to healthy and anemic controls. Further analysis, using a multivariate dynamic model of heart rate variability, suggests that the low baseline parasympathetic activity in these SCD subjects is more likely to be due to impaired respiratory-cardiac coupling, rather than the decreased cardiac baroreflex sensitivity (Figure 6). On the opposite side of the spectrum, the subset of subjects with augmented cardiac parasympathetic activity (elevated HFP_RRI,n_) but low vascular variability (low LFP_PPGa_) at baseline tended to have C-phenotype response to HUT (increased heart rate without concurrent peripheral vasoconstriction). It is possible that subjects with low baseline vascular variability would have low sympathetic modulation of peripheral resistance but high sympathetic tone, because their vessels are already largely constricted. As such, dynamic modulations of sympathetic input will not have much effect in eliciting further peripheral vasoconstriction, such as during HUT in this subset of subjects.

### Abnormal Autonomic Activity and Vasoreactivity in SCD

Imbalance between parasympathetic and sympathetic activity in SCD has been reported in multiple studies (Sangkatumvong et al., 2011; L’Esperance et al., 2013; Chalacheva et al., 2015, 2017; Khaleel et al., 2017). Our research group previously found that SCD subjects had stronger parasympathetic withdrawal to transient hypoxia (Sangkatumvong et al., 2011) and blunted cardiac baroreflex to cold face stimulation (Chalacheva et al., 2015). Others found relative sympathetic dominance in SCD (Mestre et al., 1997; Pearson et al., 2005). Martins et al. (2012) investigated HUT responses in SCD, iron deficiency anemic and healthy subjects and found also that SCD subjects did not have a large increase in heart rate compared to other subject groups, consistent with our results. We also showed previously that SCD subjects had higher frequency of vasoconstriction in response to sigh (Sangkatumvong et al., 2011), a reflex which is sympathetically mediated (Bolton et al., 1936). Similarly, another group reported that SCD subjects had stronger vasoconstriction in response to inspiratory breath hold, also a sympathetically mediated stimulus, compared to controls (L’Esperance et al., 2013). In our recent studies, we found that SCD subjects had stronger vasoreactivity to heat pain compared to controls (Chalacheva et al., 2017; Khaleel et al., 2017). These previous findings all point toward higher tendency to having peripheral vasoconstriction in response to autonomic stimuli in SCD. However, our current study suggests that the peripheral vasoconstriction responses to HUT are not different between SCD and non-SCD subjects. Moreover, further investigation using model-based analysis of baseline peripheral vascular variability found no differences in sympathetic vascular baroreflex gain among the different autonomic phenotypes in SCD subjects. On the other hand, it should be pointed out that peripheral vascular variability may not be influenced only by sympathetic nerve inputs but also vasoactive substances, such as nitric oxide and endothelin-1 (Kinlay et al., 2001). Thus low nitric oxide bioavailability, a common pathological condition in SCD (Reiter et al., 2002), could have negative impact on vascular variability. Bourque et al. (2011) suggested that nitric oxide tonically inhibits the vasoconstrictive effect of endothelin-1. With imbalance between these two mediators, endothelin-1 actions become dominant when there is low nitric oxide bioavailability, resulting in sustained vasoconstriction.

The key findings that have emerged from this study are that: (a) P-phenotype responses (peripheral vasoconstriction without significant heart rate increase) were found overwhelmingly in SCD subjects, and (b) the SCD subjects with P phenotype had lower baseline cardiac parasympathetic activity than those who displayed typical HUT responses (CP phenotype). The latter finding is consistent with previous reports that SCD subjects tend to have impaired parasympathetic activity. Further investigation using model-based analysis suggests that the low cardiac parasympathetic activity in the P-phenotype SCD subjects was due to impaired respiratory-cardiac coupling rather than decreased baroreflex gain. The physiological mechanisms underlying this finding remain to be elucidated. On the peripheral vascular control side, we could not detect any difference in baseline vascular variability in SCD subjects among HUT phenotypes. The autonomic indices of SCD subjects derived from the peripheral vascular control model also did not show any difference among HUT phenotypes. However, it should be emphasized that the lack of differences in baseline vascular variability across subject groups or phenotypes does not necessarily imply that there were no differences in sympathetic vascular tone (Malpas, 2002).

### Role of Anemia

Anemia is another significant predictor of the autonomic response to HUT. The body compensates for anemia by increasing cardiac output in order to maintain oxygen delivery. Chronic anemia leads to chronic dilation of the left ventricle in SCD (Lester et al., 1990; Gladwin and Sachdev, 2012). At the same time, anemic patients tend to have low peripheral resistance (Metivier et al., 2000). These alterations in cardiac output, oxygen delivery and peripheral resistance have direct effects on autonomic control of cardiovascular system. In this study, we found that anemia decreased the probability of having dual cardiac and peripheral vascular responses to HUT (CP phenotype) in both SCD and non-SCD subjects (Figure 5A). However, anemia and low vascular variability strongly predicts subthreshold response to HUT (ST phenotype) in SCD patients. Martins et al. (2012) reported that SCD and anemic controls had lower increase in DBP than normal controls during HUT, when hemoglobin level in SCD and anemic controls were comparable. Their finding suggests that anemia lessens the ability to vasoconstrict in response to orthostatic stress, which explains why those subjects could not sufficiently vasoconstrict to increase DBP.

### Clinical Implications for SCD

How is all this relevant to SCD? We know that decreased regional blood flow from any cause will increase the likelihood of deoxygenated HbS polymerizing within the microvasculature before they can escape to larger diameter vessels, triggering vaso-occlusion. Thus, we speculate that the P-phenotype subset of SCD subjects with prolonged peripheral vasoconstriction, but without compensatory changes in heart rate, in response to HUT would be more likely to have reduced microvascular blood flow, and thus have the highest risk of VOC. The low baseline levels of parasympathetic activity in these subjects may explain the inability of the heart to respond sufficiently to stimuli that activate the sympathetic nervous system. Under conditions of decreased peripheral vascular flow, the transit time of red blood cells through the capillary beds would be elevated, increasing the probability of rigid sickle red cell entrapment in the microvasculature and subsequent VOC. This could explain why SCD subjects tend to associate stress, cold and pain as factors that trigger VOC. A recent study has shown that hypnosis lessened the vasoconstrictive effect during pain and anticipation to pain (Bhatt et al., 2017), suggesting that interventions that reduce sympathetic drive can potentially alleviate the risk of VOC. It is possible that ST-phenotype SCD subjects may also be at increased risk for VOC because they are already peripherally vasoconstricted and, at the same time, unable to raise cardiac output to increase peripheral blood flow in response to orthostatic stress. But our ability to draw conclusions on the ST-phenotype subjects is severely limited by the small sample size of this category in the present study. Nevertheless, our finding of distinct autonomic phenotypes is consistent with clinical observations that the frequency and severity of pain crises can vary substantially across individuals with SCD (Coates et al., 2018).

One significant limitation of this study is that we included SCD and anemic non-SCD subjects who received regular transfusion therapy. While inclusion of these transfused subjects allows us to investigate a larger range of hemoglobin level and its effect on HUT responses, we could not look at the VOC frequency in SCD subjects as a function of HUT response phenotype. This is because VOC are generally prevented by chronic transfusion therapy. Nonetheless, there was a significantly greater probability of having P phenotype response in SCD than controls, independent of hemoglobin level.

### Future Perspectives

While our findings provide insight into which functional mechanisms predispose SCD subjects to having atypical HUT response, further investigations would enable us to pinpoint the cause of autonomic abnormalities in SCD subjects. In particular, it would be important to determine which underlying physiological mechanisms are primarily responsible for the decreased respiratory-cardiac coupling in the P-phenotype SCD subjects. For instance, could this difference be due to impairment of the cardiopulmonary receptors in the atrial wall or abnormality in neural transmission of respiratory drive to cardiac vagal efferents? As well, measurements that can delineate the separate contributions of sympathetic outflow, cardiac contractility and vasomotion will provide a more comprehensive understanding of the underlying basis of the different autonomic phenotypes.

## Conclusion

We have shown that SCD subjects are much more likely than non-SCD subjects to have impaired cardiac, but intact peripheral responses to orthostatic stress induced by HUT. These abnormal responses are associated with low baseline cardiac parasympathetic activity, independent of hemoglobin level. The classification of autonomic phenotypes based on HUT response may have potential use for predicting disease severity, guiding and targeting treatments/interventions to alleviate the risk of adverse outcomes in SCD.

## Ethics Statement

All experiments were conducted at Children’s Hospital Los Angeles (CHLA). The study protocol was approved by the Committee on Clinical Investigations (institutional review board of CHLA). In accordance with CHLA IRB policies, written informed consent or assent (for subjects < 14 years old) was obtained before participation in the study. In addition, parental consent was obtained if the subject was less than 18 years old.

## Author Contributions

TC, MK, RK, JW, and JD designed the study protocols. PC, RK, PS, and JS performed the experimental device setup. PC, RK, PS, SV, CD, and JD ran patient studies and collected the data. PC analyzed the data and wrote the manuscript. WT assisted with software used for data pre-processing. PC, MK, TC, JD, JW, PS, RK, SV, and CD interpreted the results. MK, TC, JW, and JD critically reviewed and edited the manuscript. RK, PS, SV, CD, and JS edited the manuscript.

## Conflict of Interest Statement

The authors declare that the research was conducted in the absence of any commercial or financial relationships that could be construed as a potential conflict of interest.

## References

[B1] Allen J. (2007). Photoplethysmography and its application in clinical physiological measurement. *Physiol. Meas.* 28 R1–R39. 10.1088/0967-3334/28/3/R01 17322588

[B2] Asyali M. H. Juusola M. (2005). Use of meixner functions in estimation of volterra kernels of nonlinear systems with delay. *IEEE Trans. Biomed. Eng.* 52 229–237. 10.1109/TBME.2004.840187 15709660

[B3] Babchenko A. Davidson E. Ginosar Y. Kurz V. Faib I. Adler D. (2001). Photoplethysmographic measurement of changes in total and pulsatile tissue blood volume, following sympathetic blockade. *Physiol. Meas.* 22 389–396. 10.1088/0967-3334/22/2/310 11411248

[B4] Batzel J. Baselli G. Mukkamala R. Chon K. H. (2009). Modelling and disentangling physiological mechanisms: linear and nonlinear identification techniques for analysis of cardiovascular regulation. *Philos. Trans. R. Soc. A Math. Phys. Eng. Sci.* 367 1377–1391. 10.1098/rsta.2008.0266 19324714 PMC3268216

[B5] Beene T. K. Eggers G. W. (1974). Use of the pulse monitor for determining sympathetic block of the arm. *Anesthesiology* 40 412–414. 10.1097/00000542-197404000-00023 4819097

[B6] Belozeroff V. Berry R. B. Khoo M. C. (2003). Model-based assessment of autonomic control in obstructive sleep apnea syndrome. *Sleep* 26 65–73. 10.1093/sleep/26.1.65 12627735

[B7] Berger R. D. Akselrod S. Gordon D. Cohen R. J. (1986). An efficient algorithm for spectral analysis of heart rate variability. *IEEE Trans. Biomed. Eng.* 33 900–904. 10.1109/TBME.1986.325789 3759126

[B8] Bhatt R. Martin S. Evans S. Lung K. Coates T. Zeltzer L. (2017). The effect of hypnosis on pain and peripheral blood flow in sickle-cell disease: a pilot study. *J. Pain Res.* 10 1635–1644. 10.2147/JPR.S131859 28769584 PMC5529094

[B9] Bolton B. Carmichael E. A. Sturup G. (1936). Vaso-constriction following deep inspiration. *J. Physiol.* 86 83–94. 10.1113/jphysiol.1936.sp003345 16994738 PMC1394564

[B10] Bourque S. L. Davidge S. T. Adams M. A. (2011). The interaction between endothelin-1 and nitric oxide in the vasculature: new perspectives. *Am. J. Physiol. Integr. Comp. Physiol.* 300 R1288–R1295. 10.1152/ajpregu.00397.2010 21368267

[B11] Chalacheva P. Kato R. M. Sangkatumvong S. Detterich J. Bush A. Wood J. C. (2015). Autonomic responses to cold face stimulation in sickle cell disease: a time-varying model analysis. *Physiol. Rep.* 3:e12463. 10.14814/phy2.12463 26177958 PMC4552538

[B12] Chalacheva P. Khaleel M. Sunwoo J. Shah P. Detterich J. A. Kato R. M. (2017). Biophysical markers of the peripheral vasoconstriction response to pain in sickle cell disease. *PLoS One* 12:e0178353. 10.1371/journal.pone.0178353 28542469 PMC5443571

[B13] Chalacheva P. Khoo M. C. K. (2014). Estimating the baroreflex and respiratory modulation of peripheral vascular resistance. *Conf. Proc. IEEE Eng. Med. Biol. Soc.* 2014 2936–2939. 10.1109/EMBC.2014.6944238 25570606 PMC4487769

[B14] Chandler M. P. Mathias C. J. (2002). Haemodynamic responses during head-up tilt and tilt reversal in two groups with chronic autonomic failure: pure autonomic failure and multiple system atrophy. *J. Neurol.* 249 542–548. 10.1007/s004150200062 12021943

[B15] Charlot K. Hierso R. Lemonne N. Romana M. Tressières B. Lalanne-Mistrih M.-L. (2017). Changes in autonomic nervous activity during vaso-occlusive crisis in patients with sickle cell anaemia. *Br. J. Haematol.* 177 484–486. 10.1111/bjh.14064 27009926

[B16] Coates T. D. Chalacheva P. Zeltzer L. Khoo M. C. K. (2018). Autonomic nervous system involvement in sickle cell disease. *Clin. Hemorheol. Microcirc.* 68 251–262. 10.3233/CH-189011 29614636

[B17] Davis M. J. Hill M. A. (1999). Signaling mechanisms underlying the vascular myogenic response. *Physiol. Rev.* 79 387–423. 10.1152/physrev.1999.79.2.387 10221985

[B18] Domínguez-Almendros S. Benítez-Parejo N. Gonzalez-Ramirez A. R. (2011). Logistic regression models. *Allergol. Immunopathol.* 39 295–305. 10.1016/j.aller.2011.05.002 21820234

[B19] Eaton W. A. Hofrichter J. Ross P. D. (1976). Editorial: delay time of gelation: a possible determinant of clinical severity in sickle cell disease. *Blood* 47 621–627. 1260125

[B20] Elgendi M. (2012). On the analysis of fingertip photoplethysmogram signals. *Curr. Cardiol. Rev.* 8 14–25. 10.2174/157340312801215782 22845812 PMC3394104

[B21] Everitt B. S. Landau S. Leese M. Stahl D. (2011). *Cluster Analysis*, 5th Edn Chichester: Wiley 10.1002/9780470977811

[B22] Gladwin M. T. Sachdev V. (2012). Cardiovascular abnormalities in sickle cell disease. *J. Am. Coll. Cardiol.* 59 1123–1133. 10.1016/j.jacC.2011.10.900 22440212 PMC3881188

[B23] Guyenet P. G. (2006). The sympathetic control of blood pressure. *Nat. Rev. Neurosci.* 7 335–346. 10.1038/nrn1902 16760914

[B24] James T. N. Riddick L. Massing G. K. (1994). Sickle cells and sudden death: morphologic abnormalities of the cardiac conduction system. *J. Lab. Clin. Med.* 124 507–520. 7930876

[B25] Khaleel M. Puliyel M. Shah P. Sunwoo J. Kato R. M. Chalacheva P. (2017). Individuals with sickle cell disease have a significantly greater vasoconstriction response to thermal pain than controls and have significant vasoconstriction in response to anticipation of pain. *Am. J. Hematol.* 92 1137–1145. 10.1002/ajh.24858 28707371 PMC5880319

[B26] Khoo M. C. K. (2018). *Physiological Control Systems: Analysis, Simulation, and Estimation*, 2nd Edn Hoboken, NJ: John Wiley and Sons, Inc. 10.1002/9781119058786

[B27] Khoo M. C. K. Chalacheva P. (2016). Model-derived markers of autonomic cardiovascular dysfunction in sleep-disordered breathing. *Sleep Med. Clin.* 11 489–501. 10.1016/j.jsmc.2016.07.003 28118872 PMC5270554

[B28] Kim J. M. Arakawa K. VonLintel T. (1975). Use of the pulse-wave monitor as a measurement of diagnostic sympathetic block and of surgical sympathectomy. *Anesth. Analg.* 54 289–296. 10.1213/00000539-197505000-00005 1169013

[B29] Kinlay S. Creager M. A. Fukumoto M. Hikita H. Fang J. C. Selwyn A. P. (2001). Endothelium-derived nitric oxide regulates arterial elasticity in human arteries in vivo. *Hypertension* 38 1049–1053. 10.1161/hy1101.095329 11711496

[B30] Kleiger R. E. Miller J. P. Bigger J. T. Moss A. J. (1987). Decreased heart rate variability and its association with increased mortality after acute myocardial infarction. *Am. J. Cardiol.* 59 256–262. 10.1016/0002-9149(87)90795-8 3812275

[B31] L’Esperance V. S. Cox S. E. Simpson D. Gill C. Makani J. Soka D. (2013). Peripheral vascular response to inspiratory breath hold in paediatric homozygous sickle cell disease. *Exp. Physiol.* 98 49–56. 10.1113/expphysiol.2011.064055 22660812 PMC4463767

[B32] Lester L. A. Sodt P. C. Hutcheon N. Arcilla R. A. (1990). Cardiac abnormalities in children with sickle cell anemia. *Chest* 98 1169–1174. 10.1378/CHEST.98.5.1169 2146092

[B33] Lucini D. Porta A. Milani O. Baselli G. Pagani M. (2000). Assessment of arterial and cardiopulmonary baroreflex gains from simultaneous recordings of spontaneous cardiovascular and respiratory variability. *J. Hypertens.* 18 281–286. 10.1097/00004872-200018030-00007 10726714

[B34] Malpas S. C. (2002). Neural influences on cardiovascular variability: possibilities and pitfalls. *Am. J. Physiol. Heart Circ. Physiol.* 282 H6–H20. 10.1152/ajpheart.2002.282.1.H6 11748042

[B35] Malpas S. C. Leonard B. L. Guild S. J. Ringwood J. V. Navakatikyan M. Austin P. C. (2001). The sympathetic nervous system’s role in regulating blood pressure variability. *IEEE Eng. Med. Biol. Mag.* 20 17–24. 10.1109/51.917720 11321716

[B36] Martins W. D. A. Lopes H. F. Consolim-Colombo F. M. Gualandro S. D. F. M. Arteaga-Fernández E. Mady C. (2012). Cardiovascular autonomic dysfunction in sickle cell anemia. *Auton. Neurosci.* 166 54–59. 10.1016/j.autneu.2011.07.011 21868290

[B37] Mestre J. C. R. Hernández A. Agramonte O. Hernández P. (1997). Cardiovascular autonomic dysfunction in sickle cell anemia: a possible risk factor for sudden death? *Clin. Auton. Res.* 7 121–125. 10.1007/BF02308838 9232355

[B38] Metivier F. Marchais S. J. Guerin A. P. Pannier B. London G. M. (2000). Pathophysiology of anaemia: focus on the heart and blood vessels. *Nephrol. Dial. Transplant.* 15(Suppl. 3), 14–18. 10.1093/oxfordjournals.ndt.a027970 11032352

[B39] Mitra S. K. (2006). *Digital Signal Processing: a Computer Based Approach*, 3rd Edn Boston, MA: McGraw-Hill.

[B40] Nebor D. Bowers A. Hardy-Dessources M.-D. Knight-Madden J. Romana M. Reid H. (2011). Frequency of pain crises in sickle cell anemia and its relationship with the sympatho-vagal balance, blood viscosity and inflammation. *Haematologica* 96 1589–1594. 10.3324/haematol.2011.047365 21750084 PMC3208675

[B41] ESC/NASPE Task Force (1996). Heart rate variability: standards of measurement, physiological interpretation and clinical use. Task force of the european society of cardiology and the North American society of pacing and electrophysiology. *Eur. Heart J.* 17 1043–1065. 8737210

[B42] Pagani M. Lucini D. Rimoldi O. Furlan R. Piazza S. Porta A. (1996). Low and high frequency components of blood pressure variability. *Ann. N. Y. Acad. Sci.* 783 10–23. 10.1111/j.1749-6632.1996.tb26704.x 8853630

[B43] Parati G. Di Rienzo M. Mancia G. (2000). How to measure baroreflex sensitivity: from the cardiovascular laboratory to daily life. *J. Hypertens.* 18 7–19. 10.1097/00004872-200018010-00003 10678538

[B44] Parati G. Saul J. P. Di Rienzo M. Mancia G. (1995). Spectral analysis of blood pressure and heart rate variability in evaluating cardiovascular regulation. A critical appraisal. *Hypertension* 25 1276–1286. 10.1161/01.HYP.25.6.1276 7768574

[B45] Pearson S. R. Alkon A. Treadwell M. Wolff B. Quirolo K. Boyce W. T. (2005). Autonomic reactivity and clinical severity in children with sickle cell disease. *Clin. Auton. Res.* 15 400–407. 10.1007/s10286-005-0300-9 16362543

[B46] Rees D. C. Williams T. N. Gladwin M. T. (2010). Sickle-cell disease. *Lancet* 376 2018–2031. 10.1016/S0140-6736(10)61029-X 21131035

[B47] Reiter C. D. Wang X. Tanus-Santos J. E. Hogg N. Cannon R. O. III Schechter A. N. (2002). Cell-free hemoglobin limits nitric oxide bioavailability in sickle-cell disease. *Nat. Med.* 8 1383–1389. 10.1038/nm799 12426562

[B48] Sacha J. (2013). Why should one normalize heart rate variability with respect to average heart rate. *Front. Physiol.* 4:306. 10.3389/fphys.2013.00306 24155724 PMC3804770

[B49] Sangkatumvong S. Khoo M. C. K. Kato R. Detterich J. A. Bush A. Keens T. G. (2011). Peripheral vasoconstriction and abnormal parasympathetic response to sighs and transient hypoxia in sickle cell disease. *Am. J. Respir. Crit. Care Med.* 184 474–481. 10.1164/rccm.201103-0537OC 21616995 PMC3175540

[B50] Seals D. R. Suwarno N. O. Joyner M. J. Iber C. Copeland J. G. Dempsey J. A. (1993). Respiratory modulation of muscle sympathetic nerve activity in intact and lung denervated humans. *Circ. Res.* 72 440–454. 10.1161/01.RES.72.2.440 8418993

[B51] Secomb T. W. (2008). Theoretical models for regulation of blood flow. *Microcirculation* 15 765–775. 10.1080/10739680802350112 18951240 PMC2593747

[B52] Stewart J. M. (2012). Mechanisms of sympathetic regulation in orthostatic intolerance. *J. Appl. Physiol.* 113 1659–1668. 10.1152/japplphysiol.00266.2012 22678960 PMC3524660

[B53] Thomas G. D. (2011). Neural control of the circulation. *Adv. Physiol. Educ.* 35 28–32. 10.1152/advan.00114.2010 21385998

[B54] Wang S. Randall D. C. Knapp C. F. Patwardhan A. R. Nelson K. R. Karounos D. G. (2012). Blood pressure regulation in diabetic patients with and without peripheral neuropathy. *Am. J. Physiol. Regul. Integr. Comp. Physiol.* 302 R541–R550. 10.1152/ajpregu.00174.2011 22049233 PMC3311521

[B55] Xiao X. Mullen T. J. Mukkamala R. (2005). System identification: a multi-signal approach for probing neural cardiovascular regulation. *Physiol. Meas.* 26 R41–R71. 10.1088/0967-3334/26/3/R01 15798289

[B56] Zaqqa M. Massumi A. (2000). Neurally mediated syncope. *Tex. Heart Inst. J.* 27 268–272. 11093411 PMC101078

