# Supplementary material for: Sickle Cell Disease Subjects Have a Distinct Abnormal Autonomic Phenotype Characterized by Peripheral Vasoconstriction With Blunted Cardiac Response to Head-Up Tilt
Source: Front Physiol. 2019 Apr 11;10:381. doi: 10.3389/fphys.2019.00381 (PMC6470196; doi:10.3389/fphys.2019.00381)
Supplement: Supplementary file 1 [file Table_1.DOCX]

# Supplemental materials

## S1 Determination of reference supine value

We found that the mean pre-HUT PPGa (49.1 nu) was significantly different from baseline mean (66.3 nu, p < 0.0001) while the mean post-HUT PPGa (63.4 nu) was not different from baseline (p = 0.4790). This shows that the post-HUT PPGa was more reflective of the baseline state (i.e. before any interventions), and therefore we used the post-HUT value as the reference supine value.

We found that the HUT response classification based on the pre-HUT values was consistent with the classification based on the post-HUT values (Supplemental Table 1). This additional analysis shows that the majority of subjects had a dual cardiac and peripheral response to HUT (CP-phenotype) while subjects with only peripheral vasoconstriction response to HUT (P-phenotype) were mostly SCD subjects.

Supplemental Table 1. SCD and non-SCD subjects by HUT response classification, based on pre-HUT values: count (row %)

|  | HUT phenotypes | | | |  |
| --- | --- | --- | --- | --- | --- |
|  | CP | C | P | ST | Total |
| SCD | 10 (37.0) | 4(14.8) | 10 (37.0) | 3 (11.1) | 27 |
| Non-SCD | 28 (71.8) | 7 (18.0) | 4 (10.3) | 0 (0) | 39 |
| Total | 38 | 11 | 14 | 3 | 66 |

The contingency table shows the number of SCD and non-SCD subjects in different HUT phenotypes (likelihood ratio χ^2^ = 14.3, p = 0.0025). CP = having both cardiac and peripheral responses; C = having only cardiac response, P = having only peripheral response; ST = having subthreshold cardiac and peripheral response.

Supplemental Table 2. Blood parameters by hemoglobinopathy

|  | Healthy | SCT | HS | Anemic non-SCD | SCD | P-value |
| --- | --- | --- | --- | --- | --- | --- |
| N | 11 | 8 | 7 | 13 | 27 | - |
| Hemoglobin (g/dL) | 13.1 (0.4) | 13.8 (0.5) | 13.8 (1.1) | 9.5 (0.3)^a,b,c^ | 9.5 (0.3)^a,b,c^ | <0.0001 |
| Hematocrit (%) | 40.4 (1.3) | 40.0 (1.2) | 38.9 (2.8) | 31.9 (1.0)^a,b,c^ | 27.7 (1.0)^a,b,c^ | <0.0001 |
| Reticulocyte^*^ (%) | 1.17 (1.2) | 1.55 (1.4) | 3.13 (13.2) | 3.07 (7.8) | 9.02 (9.9)^a,b,d^ | <0.0001 |
| Plasma hgb^*^ (mg/dL) | 25.2 (5.0) | 29.4 (20.8) | 49.8 (50.9) | 96.1 (61.1)^a,b^ | 71.9 (63.5)^a,b^ | <0.0001 |
| Free heme^*^ (μM) | 0.07 (0.11) | 0.12 (0.14) | 0.14 (0.93) | 2.32 (1.20)^a,b,c^ | 1.26 (1.23)^a,b,c^ | <0.0001 |
| Hemopexin^*^ (μg/mL) | 473.4 (168.4) | 452.4 (370.4) | 486.0 (261.3) | 9.6 (16.1)^a,b,c^ | 310.2 (333.2)^a,d^ | <0.0001 |

Healthy: healthy AA; SCT: sickle cell trait AS; HS: hereditary spherocytosis; anemic non-SCD: beta thalassemia major, hemoglobin H and hemoglobin H constant spring; SCD: homozygous SS, S-β0 thalassemia and S-β+ thalassemia. Normally distributed data are shown as mean (standard error of mean) with p-value from ANOVA. Non-normally distributed data, indicated by ^*^, are shown as median (interquartile range) with p-values from Kruskal-Wallis test. Superscript letters indicate significant pairwise difference (p < 0.05) from healthy (a), SCT (b), HS (c) and anemic non-SCD (d).
